# Agency via Life Satisfaction as a Protective Factor From Cumulative Trauma and Emotional Distress Among Bedouin Children in Palestine

**DOI:** 10.3389/fpsyg.2019.01674

**Published:** 2019-07-23

**Authors:** Guido Veronese, Alessandro Pepe, Federica Cavazzoni, Hania Obaid, Jesus Perez

**Affiliations:** ^1^Department of Human Sciences and Education, University of Milano-Bicocca, Milan, Italy; ^2^International University of La Rioja, Ciencias de la Salud, Logroño, Spain

**Keywords:** agency, life satisfaction, psychological trauma, children, war

## Abstract

Adopting an ecological perspective on children’s functioning and psychological well-being, we investigated the association between agency and life satisfaction, and its bearing on trauma symptoms and negative emotions in a group of Bedouin children living in the occupied Palestinian territories. Specifically, we hypothesized that the more children were agentic, the more they would be satisfied with their lives; and that greater life satisfaction would be associated with better affect balance, and reduced trauma symptoms. A sample of 286 Bedouin children attending primary schools in four different villages in the Jordan Valley completed the *multidimensional students’ life satisfaction scale (MSLSS)*, *positive affect and negative affect scale for children (PANAS-C), Children’s Impact of Event Scale (CRIES-13), and the children’s hope scale (CHS).* Structural equation modeling was performed to evaluate the cumulative network of direct and indirect effects between children’s agency, life satisfaction, and trauma symptoms. The findings confirmed the key role of life satisfaction in mitigating traumatic reactions. Higher levels of life satisfaction were associated with reduced negative emotions and trauma symptoms, suggesting that agency may be viewed as a pre-determining factor with the potential to protect children from trauma symptoms. We discuss the implications for research and clinical practice.

## Introduction

Children who grow up exposed to war and military violence in contexts of ongoing structural oppression are known to be at risk of psychological burden caused by cumulative exposure to traumatic events in unsafe environments (Cook et al., 2017; Manzanero et al., 2018). Numerous studies have examined the psychological consequences, and effects on children’s mental health, of being exposed to the violence of war and military occupation (Catani, 2018). These works offer quantitative evidence of the association between exposure to violence and negative mental health outcomes (Rabaia et al., 2014). A smaller body of studies on the other hand have associated exposure to adverse events with positive outcomes such as: positive coping styles; personal growth; life satisfaction; social; and self-competence (Barber and Schluterman, 2009). More recently, research has begun to focus on functioning and protective factors that enable children to conserve their psychological wellbeing in the face of ongoing traumatic events (Boyden, 2003; Veronese et al., 2012b, 2017a; Masten, 2017). Studies conducted from the last-mentioned perspective view children as competent and socially situated actors who are capable of actively mobilizing sources of agency to protect themselves from the negative consequences of war and violence (Gilligan, 2009; Marshall, 2014; Veronese et al., 2017a, 2018). This line of inquiry has focused on the socio-ecological factors that can protect children from trauma and foster their resilience (Betancourt and Khan, 2008; Diab et al., 2018b). The most influential determinants of resilience in children exposed to ongoing cumulative trauma are thought to include: family and parental functioning; personal resources; socio-economic, household, and environment-related factors; education; and social and community support (Masten, 2001; Fazel et al., 2012).

Life satisfaction and positive affect balance have been associated with enhanced mental health and reduced post-traumatic symptoms in contexts such as Palestine that are marked by chronic conflict and ongoing military violence (Veronese et al., 2012a, 2018; Veronese and Pepe, 2018). Veronese and Pepe (2017a) found that a clinically referred group of Palestinian children reported poorer life satisfaction and higher levels of trauma than peers referred to non-clinical psycho-social interventions who were characterized by higher life satisfaction and lower levels of trauma symptoms. The authors found that children’s perceived life satisfaction in the domains of family and school was the main discriminant between the clinically referred and the community-referred children. Another study with youths exposed to war in Israel showed that life satisfaction and negative affect, but not positive affect, mediated the relationship between gratitude and PTSD symptoms. The strongest significant predictor of PTSD symptoms in this case was life satisfaction, which the authors suggested may be the cognitive pathway through which gratitude is associated with PTSD (Israel-Cohen et al., 2015). Finally, Black South African adolescents were more satisfied with their lives when able to mobilize resilience resources following traumatic experiences (Botha and Van den Berg, 2016). All of the cited research found that children’s capacity to maintain a sense of satisfaction with their lives had the effect of protecting their mental health, providing them with resources for survival even when their general life conditions were severely compromised (Getanda et al., 2015). Yet these studies did not explain by what means life satisfaction is sometimes preserved, mitigating trauma symptoms, and enhancing psychological functioning. One possibility is that human agency plays a role in sustaining life satisfaction in children growing up amidst extremely adverse living conditions (Gilligan, 2009; Alexander et al., 2015). Agency is a sociological construct that has been extensively explored in philosophy and the social sciences, especially in relation to at-risk, and marginalized populations (Shakya and Rankin, 2008; Coe and Jordhus-Lier, 2011).

Bandura (1998, 2001) operationalized the construct of human agency as individuals’ personal resources such as self-confidence and self-efficacy. The concept of personal resources had earlier been developed by Hobfoll (1988), who in his conservation of resources theory, defined them as qualities that people possess, including self−esteem, self−mastery, and a sense of success. According to Bandura, self-efficacy beliefs are especially crucial to intrinsic motivation. To use his own words: “among the mechanisms of agency, none is more central, or pervasive than people’s beliefs in their efficacy to manage their functioning and to exercise control over events that affect their lives” (Benight and Bandura, 2004, p.1131). In keeping with Bandura’s theory, in a study by Slobodin et al. (2011) with a group of in-service soldiers of Bedouin ethnicity, a lack of personal resources (e.g., self-esteem and self-mastery) was found to be associated with higher levels of traumatic stress symptoms.

Likewise, in sociology, anthropology, and human geography, agency has been defined as the capacity to act positively across space and time with respect to oppressive structures in one’s environment (Jeffrey, 2012). Accordingly, agency is activated and mobilized in the home, at school, and on the streets.

Stoecklin (2018) has used the term “situated agency” to signify the construct of agency as it obtains in specific contexts and social ecologies. “Contexts are the fields where agency can be observed. Agency itself has no existence *per se*” (Stoecklin, 2018; p. 560). Accordingly, social cognitive theory rejects any duality between individual agency and its socio-ecological dimensions (Bandura, 2018). Rather, it assumes that individuals contribute to creating social systems, which in turn organize and influence their lives, giving rise to a network of reciprocal relations (Schoon and Lyons-Amos, 2017). In psychology, interactions between the person and social structures are often used to explain the human (Eccles, 2008). A socio-ecological approach to agency entails investigating how multiple layers of social structures affect individual well-being and functioning, and how different aspects of agency can shape the selection of distinct life domains (e.g., school, living environment, family, and social relations) conceptualized as ecological niches.

Although some psychological research has attempted to draw on the construct of agency to explore children’s functioning, more remains to be done to operationalize a concept that, to a certain extent, remains volatile, and vague in meaning (Alexander et al., 2015; Denov and Akesson, 2016; Veronese et al., 2018). Qualitative studies carried out in Palestine found that children are capable of re-shaping their real-life experience, actively exerting control over multiple domains of their lives such as school, social and family relations, and even their overall life context, thereby enhancing their sense of competence, and psychological wellbeing (Marshall, 2014; Denov and Akesson, 2016; Veronese et al., 2017a,b, 2018). Similarly, studies informed by social cognitive theory have found that perceived self-efficacy in coping is associated with better recovery from traumatic experiences (Benight and Bandura, 2004). Scholars advocating an “agentic” perspective on adaptation suggest that positive functioning and well-being seem to rely more on personal and social enablement than on environmental factors (Benight and Bandura, 2004).

The first challenge encountered when seeking to quantitatively investigate agency from a psychological perspective is identifying appropriate standardized instruments for measuring the construct. The fact that the construct itself has yet to be clearly conceptualized has meant that to date no self-report measures have been developed for the quantitative assessment of agency in children. Adler et al. (2008) experimented with an indirect method of assessing agency by using a rating scale to measure how much responsibility clients described taking for their lives in a retrospective narrative account of a course of psychotherapy they had just completed. For our present research purposes, we found Snyder’s conceptualization and operationalization of hope to offer the best fit with our own definition of agency. Within Snyder’s overall construct of hope, agency is understood as both a state and a dispositional trait that interacts with children’s ability to harness their motivation, resulting in enhanced personal wellbeing and greater ability to cope with hardship and adversity (Snyder et al., 1991, 1996). More specifically, hope is defined as a positive mental state resulting from belief in one’s own capacity to initiate and sustain actions for achieving goals (agency), and one’s self-perceived ability to generate ways of achieving one’s goals (pathways).

## The Study

### Aims and Scope

In our previous research in Palestine, we found that children maintained good subjective well-being and life satisfaction, even in the face of ongoing, and cumulative trauma (see Veronese et al., 2012a, 2017a). More specifically, we developed and tested a structural model in which life satisfaction acted as a predictor of trauma via negative emotions. To date, however, no quantitative study has investigated how children conserve life satisfaction in war-torn environments, although, previous qualitative research suggests that agency may be a strong contributor to life satisfaction (Veronese and Castiglioni, 2015; Veronese et al., 2017b, 2018). Despite the challenges associated with operationalizing the construct of agency, in our pilot study we set out to examine the association between agency and life satisfaction.

More specifically, in light of the theoretical considerations just outlined, and drawing on an ecological perspective on children’s functioning and psychological wellbeing, we set out in the current study to investigate the relationship between agency and life satisfaction, and the latter’s role in mitigating trauma-related symptoms and negative emotions, in a group of Bedouin children living in the West Bank, in the occupied Palestinian territories.

More specifically, we hypothesized that the more children were agentic, the more they would be satisfied with their lives (H1). We further predicted that greater life satisfaction would be associated with reduced trauma symptoms (H2). Hence, we expected the relationship between agency and trauma symptoms to be mediated by life satisfaction. Gender and age were included in the research design as control variables.

### Bedouin Children in Palestine

The Bedouin are a large minority group with a semi-nomadic lifestyle: They are mainly concentrated in the Negev region of Southern Israel, where they number about 280,000 (Central Bureau of Statistics, 2013), and the Jericho Region of the West Bank, home to 40,000 Bedouin who are mainly refugees from the Negev desert (Massad et al., 2017). Israel currently recognizes the Bedouins as a minority group and has authorized the Negev Bedouins to settle in a restricted area that comprises the city of Rahat and six other towns. About 50,000 Bedouins have moved to this area with the encouragement of the state (Knesset, 2019). The rest of the Bedouin population in the Negev live in dozens of unrecognized villages with no municipal status, where they face demolition orders, and suffer discrimination on ethnic grounds (Meir, 2005). The destruction of their homes, a lack of basic supplies including electricity and clean water, food insecurity, the confiscation of land, and disrupted school attendance have deprived the Bedouin children of their basic rights, including the right to harmonious development (Mihlar, 2011). In addition, thirty-five unrecognized Bedouin villages have been declared illegal by Israel. As a result, the Bedouin population lives under constant threat of deportation and temporary relocation in limited zones of Area C which is controlled by the Israeli army and governed by the Palestinian Authority (United Nations Relief and Works Agency for Palestine Refugees in the Near East [UNRWA], 2013).

Research has confirmed that these ongoing precarious living conditions and traumatic events have severely compromised the psychological functioning of Bedouin children, in terms of both impaired mental health and trauma-related syndromes. In a study by Massad et al. (2017), 44% of a sample of children had been diagnosed with psychiatric disorders related to traumatic experience and cumulative trauma. Similarly, Shaik Muhammad et al. (2017) found that Bedouin children in Israel, especially boys, displayed emotional and behavioral issues as a direct consequence of trauma, and exposure to violence. Finally, Bedouin adolescents living in unrecognized villages and exposed to the demolition of homes were found to be less capable of mobilizing coping resources, and more at risk of stress reactions and anxiety than adolescents living in recognized villages (Braun-Lewensohn et al., 2014; Al-Said et al., 2018). To the best of our knowledge, no studies to date have focused on Bedouin children’s ability to cope with trauma or their agency in mobilizing resources to enhance their personal wellbeing and resilience. The present exploratory study is intended to fill this gap in the scientific literature by modeling how children can control their traumatic distress via an “agentic” and active disposition that boosts their satisfaction with life across multiple ecological domains such as their relations with family, peers, school, and living environment.

## Materials and Methods

### Context

Following the 1995 Oslo II Accord, the West Bank was divided into areas A and B, both officially controlled by the Palestinian Authority, and Area C, where Israel has full control over security and civil affairs (United Nations Relief and Works Agency for Palestine Refugees in the Near East [UNRWA], 2013). The Jordan Valley and northern Dead Sea constitute almost 30 percent of the West Bank and 90% percent of this region has been designated as Area C. In this area, Israel impedes the development of adequate housing, infrastructure, and livelihoods for the Palestinian communities, preventing them from entering or using about 85% of the territory (United Nations Office for the Coordination of Humanitarian Affairs [OCHA], 2017). Most of Area C is off-limits to Palestinians, being classified as settlement areas, firing zones, or natural reserves. According to B’tselem (2018), reports the Israeli civil administration demolished at least 698 Palestinian residential units in the Jordan Valley between January 2007 and September 2017: 2,948 Palestinians (including 1,334 minors) have lost their homes (283 adults – and 386 minors – at least twice). The homes of most Palestinians living in these areas are not connected to water and electricity networks, and construction is heavily restricted. Moreover, nearly a third of the residential areas in Area C lack primary schools, forcing the children to travel long distances to school, exposing them to harassment by Israeli settlers, and requiring them to pass military checkpoints (United Nations Office for the Coordination of Humanitarian Affairs [OCHA], 2017).

### Participants

The participants in our pilot study were 286 Bedouin children attending primary schools in four different villages in the Jordan Valley, Area C, West Bank (29.3% from Deouq Al-Fauqa, 7,7% from Fasayel, 21% from Badou Ka’abneh, and 42%, from Al Khan Al-Ahmer). Children’s ages ranged from 7 to 16 years (*M* = 12.02; *SD* = 2.05); 65 were males (45.5%); and 78 were females (54.5%). All participants were of Muslim religion and Bedouin ethnicity; the children’s ages ranged from 9 to 16 years. Inclusion and exclusion criteria were assessed via clinical interview by specialized psychologists and counselors working at the educational institutions that took part in the research. To be included in the study, participants were required not to have been diagnosed with physical or psychological syndromes and to be in good health. Children were excluded from the study if they met diagnostic criteria or were currently receiving treatment for PTSD (7% of the sample, *n* = 10). Furthermore, only children who had been directly exposed to one or more episodes of violence or had witnessed violent acts over the 2 months prior to the study were included in the sample. All children met this last criterion. Indeed, all participants were referred by their school principals because they had either been exposed to violent episodes such as military incursions or settler attacks on their villages (80%) or were currently displaced following demolition of their homes (20%).

### Instruments and Procedures

The self-report questionnaires were administered to the children in their classrooms during school hours. Local social workers were trained to individually interview the children and complete the research instruments based on their responses. The children and their families had been fully informed of the research aims and were aware that they could decline to complete the questionnaires (or to answer specific items) or withdraw from the study at any time. Written informed consent was obtained from the children and their parents. During the administration of the questionnaire, the children were provided with all necessary clarification concerning the meaning of the questions. The questionnaires were anonymized by coding the children’s names to protect confidentiality. The study was approved by the Ethics Board of the University of Milano-Bicocca (Prot. No. 368) and complied with the ethical guidelines drawn up by [American Psychological Association [APA], 2010].

The following measures were administered:

#### Multidimensional Students’ Life Satisfaction Scale (MSLSS) (Huebner and Dew, 1996)

This scale includes 40 items exploring life satisfaction across five specific dimensions: family, friends, school, living environment, and self (Huebner, 2004). The items are rated on a Likert scale: never = 1; sometimes = 2; often = 3; almost always = 4 (e.g., I like being in school; school is interesting. I like where I live, I like my neighborhood). This instrument provides a subjective measure of individuals’ global cognitive judgements of satisfaction with their lives (Diener, 1984; Pavot et al., 1991). The cross-cultural adaptation, psychometric proprieties, and factor structure of the Multidimensional student life satisfaction scale (MSLSS) had already been evaluated in a previous study with Palestinian children (Veronese and Pepe, 2018). Thus, in the present study, the MSLSS was administered in its Arabic-language adapted and validated version (Veronese and Pepe, 2018), which measures measuring only four out of the original five dimensions of children’s life satisfaction (specifically: family, friends, school, and living environment). The factor “Myself” was excluded from the Arabic validated version of the instrument because it did not display an acceptable level of internal consistency (see Veronese and Pepe, 2018).

#### Positive Affect and Negative Affect Scale for Children (PANAS-C)

This scale is the child’s brief version of the positive and negative affect schedule (Watson et al., 1988) and it comprises 10 items, five measuring positive affect, and five negative affect. Children can express how much they have recently experienced each emotion via a 5-point Likert scale, ranging from 1 (not at all) to 5 (very much). Negative and positive affectivity are understood as broad temperamental factors. The first is related to feelings of sadness, fear, guilt, and anger (Ebesutani et al., 2011) while the second “reflects the extent to which a person feels enthusiastic, active, and alert” (Watson et al., 1988, p.1063). The cross-cultural adaptation, psychometric proprieties, and factor structure of the *PANAS-C* had also been previously evaluated with Palestinian children (Veronese and Pepe, 2017b).

#### Children’s Impact of Event Scale (CRIES-13)

The Impact of Event Scale developed by Horowitz et al. (1979) measures traumatic responses in people who have experienced traumatic events. The present version (the revised children’s impact of event scale) is an adapted version that has been specifically developed for Arabic-speaking cultures. It includes three sub-scales: four items measuring intrusion (i.e., when the traumatic event is persistently re-experienced via unwanted upsetting memories, nightmares, flashbacks, emotional distress, or physical reactivity; e.g., “Do you ever find yourself thinking about the shocking event without meaning to”?); four items measuring avoidance (avoidance of trauma-related stimuli such as trauma-related thoughts and feelings or external reminders of the trauma; e.g., “Do you ever wish you could erase the event that shocked you from your memory?”) and five items measuring arousal (that is to say, the perception and processing of potentially threatening information; e.g., “Do you ever have difficulty concentrating”?). All items are rated on a 4-point scale. Interestingly, a two-dimensional (i.e., avoidance and intrusion/hyperarousal) IES-13 measurement model has been proposed by researchers concerned with the theoretical aspects of trauma (Dyregrov et al., 1996; Smith et al., 2003).

Although we accept and agree with the DSM V’s (2013) effort to base the diagnosis of posttraumatic stress disorder (PTSD) on objective criteria, rather than on subjective perceptions of stress, the peculiar characteristics of our own research sample must be taken into account. In the present study, children were assessed and diagnosed – via clinical interview – using objective criteria as recommended by the DSM V (American Psychiatric Association [APA], 2013) before being selected to take part in the research. However, because we opted to include non-clinical children only in our research design, we judged that it was appropriate to use the CRIES-13 questionnaire to assess their current subjective perceptions of threat to their lives, taking their scores as reliable indicators of self-perceived trauma. We chose to conduct the study with non-clinical children because we had concerns about the reliability of PTSD diagnoses in situations of ongoing trauma in which it is clear that there is no “post” (Altawil et al., 2018). Hence, while the children in the current sample reported their subjective perceptions of traumatic experience, we are committed to using objective criteria to assess trauma symptoms in our research program.

#### Children’s Hope Scale (CHS)

The CHS is based on a conceptualization of hope as comprising two factors: agency and pathways (Snyder et al., 1997a). It is a six-item self-report measure for second-grade children and older. Three items assess children’s agentic thoughts (self-perceived capacity to begin and continue moving toward their goals; e.g., “I think I’m doing pretty well”) and three items evaluate their thinking about pathways (confidence that they can generate routes toward achieving their goals; e.g., “I can think of many ways to get the things in life that are most important to me”) (Snyder et al., 1997b). Items are rated on a 5-point scale of frequency: 0 = none of the time, 1 = a little of the time, 2 = some of the time, 3 = most of the time, and 4 = always. For the purposes of the present research, we took *agency and pathways* together as a combined measure of children’s sense of agency, rather than as subcomponents of the construct of hope.

Finally, in order to generate a list of the potentially frightening events typically experienced by the children in their everyday lives, and in the absence of a standardized trauma checklist, each respondent was asked to write about, or draw something that had particularly terrified him/her during the previous 2 months. We chose not to use standardized traumatic checklists because those we found in the literature were designed to record generic war events that did not reflect the complexity of the specific ongoing traumatic situation in which our participants were immersed. Hence, the inductive method we adopted enabled us to focus on frightening events in the specific setting of Bedouin villages. It also provided us with richer insight into the children’s perspectives on their experience, and individual differences in how they perceived their traumatic life circumstances. The children’s representations of traumatic events were content analyzed and categorized by the researchers using the discussion and consensus method. To minimize subjective interpretation by the interviewers, the children were asked to comment on and explain their own drawings. The written materials were translated into English by a bilingual assistant researcher and content analysis was applied to the texts.

### Data Analysis and Modeling Strategy

**FIGURE 1:**
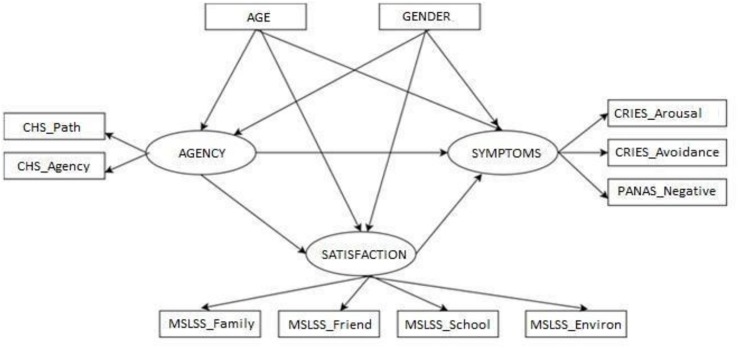
Conceptual model of pathways connecting latent variables and observed indicators. Arrows indicate direct effects between variables; ellipses indicate latent variables; and rectangular boxes represent observed variables. Age and gender were modeled as covarying variables.

In order to evaluate the cumulative network of direct and indirect effects between children’s agency, life satisfaction, and trauma symptoms, structural equation modeling (SEM) techniques (for details see Bollen, 1989) were applied. The conceptual model comprised three latent variables (i.e., children’ sense of agency, life satisfaction, and trauma symptoms), nine measured indicators, and two manifest exogenous controlling variables (i.e., children’s age and gender) (see Figure 1).

First, the latent variable “sense of agency” was assessed via the observed variables: *agency* and *pathway*. Existing research (Wessells, 2016; Veronese et al., 2018) suggests that agency functions as a resource for children who have experienced trauma, helping them to deal with negative everyday events. Next, trauma symptoms – the target latent variables – were measured via the observed variables: *intrusion/hyper-arousal* and *avoidance* (CRIES-13), and *negative affect* (PANAS-C). More specifically, the items in the Arabic version of the CRIES-13 assessed two categories of trauma symptoms described in the diagnostic and statistical manual of mental disorders, fifth edition (DSM-V, American Psychiatric Association [APA], 2013): criteria C (avoidance) and D (arousal); while for purposes of the current study, we also included children’s negative affect as a third manifest indicator of the latent variable. The addition of negative affect was in keeping with current theoretical frameworks that associate specific negative emotions and DSM-5 criteria for PTSD (Brown et al., 2016; Badour et al., 2017), positing that negative emotions play a key role in trauma and should therefore be taken into account in trauma research, and the clinical treatment of trauma symptoms. Furthermore, the inclusion of an additional manifest indicator stood to increase the robustness of the measurement model (Kline, 2016). Consistently with previous investigations (Norris, 1992; Veronese and Pepe, 2018), the symptoms of trauma were modeled as the target variable in the conceptual model. Finally, a third latent variable, life satisfaction, was estimated by measuring four dimensions of the Multidimensional Student Life Satisfaction Scale (namely, satisfaction with family, friends, school, and environment). As recommended in the literature (Stiglitz et al., 2009), the construct of life satisfaction was operationalized as children’s satisfaction with multiple key life domains. Finally, age and gender were included as controlling exogenous observed variables and their effects on all the latent variables were estimated.

The statistical significance of the model was evaluated by calculating its goodness of fit. Goodness-of-fit indices indicate the degree to which a conceptual model overlaps with empirical data, thus providing a basis for interpreting the results of the structural equation model. In the present study, the following fit indices were adopted: chi-square (χ2) for which a statistically non-significant value indicates a meaningful degree of overlap between the two matrices (Σ and S) (Schumacher and Lomax, 1996); normed chi-square (NC), or χ2 divided by the model’s degrees of freedom, for which values of 1.0–3.0 suggest good fit (Carmines and McIver, 1981); root-mean-square-residual error of approximation (RMSEA), or the square root of the mean square residuals between Σ and S (Steiger and Lind, 1980), for which values of 0.08 or lower indicate good fit (Marsh and Hau, 2014), with more robust models requiring a 90% confidence interval and a cut-off value of 0.07 (MacCallum et al., 1996); standardized root mean square residual (SRMR, 0.05) (Marsh and Hau, 2014); normed fit Index (NFI; 0.95) (Morin et al., 2013); Tucker–Lewis Index (TLI; 0.95) (Morin et al., 2013).

In order to identify and skip multivariate outliers, all variables were preliminarily checked by computing Mahalanobis’ distance (*p* < 0.001). Three extreme multivariate values were omitted from the analysis. Next, the data were assessed to verify whether the scores were normally distributed. Given that none of the variables under study displayed kurtosis or skewness values exceeding the recommended limits [−2, +2; George and Mallery, 2010], the Maximum Likelihood method (Graham, 2006) was adopted to estimate the parameters for the SEM analysis.

## Results

Categorization of the children’ s self-reported traumatic experiences showed that approximately half of the interviewees reported having been exposed to more than one event related to political violence. About one-third (32.5%) described traumatic experiences involving military violence (such as house demolitions, incursions by the Israeli army, exposure to military drones, sound-bombs, tear gas, and shootings); 17.4% self-reported episodes were connected with the Israeli occupation (e.g., being threatened by settlers or the Israeli police). In addition, a considerable percentage of children (32%) reported that they were afraid of wild animals (such as snakes, dogs, scorpions, or hyenas) or frightened because of the unsafe environment in their village (5.2%).

Finally, five percent of children had experienced episodes involving community and/or family violence (such as sexual harassment, threat, or physical violence). Finally, 4.1% reported having nightmares and experiencing fear due to having watched horror movies (see Table 1).

**TABLE 1:** Frightening events check-list.

**Traumatic event**	**Number of mentions**		
	**Male (%)**	**Female (%)**	**Total (%)**
Military violence *House demolitions*, *Israeli army incursions*, *drones*, *sound-bombs*, *tear gas*, and *shootings*	17 (30)	39 (70)	56 (32.5)
Israeli occupation *Presence of settlements*, *settlers*, and *Israeli police*	15 (50)	15 (50)	30 (17.4)
Unsafe environment *Road*, *streets*, and *route to school*	8 (89)	1 (11)	9 (5.2)
Community and/or family violence *Sexual harassment*, *rape*, and *physical violence*	3 (33)	6 (67)	9 (5.2)
Wild animals *Snakes*, *scorpions*, *dogs*, and *hyenas*	25 (45)	30 (55)	55 (32)
Horror movies *Ghosts* and *killer dolls*	2 (29)	5 (71)	7 (4.1)

Table 2 offers a summary of the main descriptive statistics for all the variables included in the study, along with their internal consistency coefficients.

**TABLE 2:** Main descriptive statistics for quantitative measures.

	**Min**	**Max**	***M***	***SD***	**Skewness**	**Kurtosis**	**α**
1. Agency	3.00	15.00	11.98	3.03	–0.98	0.02	0.76
2. Pathway	3.00	15.00	11.94	2.95	–0.98	–0.65	0.71
3.Satisfaction w. Family	4.00	20.00	14.42	2.08	–1.58	1.65	0.74
4. Satisfaction w. Friends	4.00	20.00	13.36	2.56	–0.69	0.14	0.56
5. Satisfaction w. School	3.00	15.00	10.31	2.40	–1.35	–0.19	0.77
6. Satisfaction w. Environment	3.00	15.00	10.69	2.24	–0.60	1.81	0.53
7. Intrusion/Hyper-arousal	4.00	20.00	9.31	3.27	0.16	–0.16	0.73
8. Avoidance	5.00	25.00	11.33	3.89	0.24	–0.79	0.67
9. Negative affect	5.00	25.00	12.76	4.47	0.31	–0.55	0.77

*Standard error = 0.14, α, nternal consistency.*

The results of the correlational analysis are reported in Table 3.

**TABLE 3:** Zero-order correlation among variables under study.

	**1**	**2**	**3**	**4**	**5**	**6**	**7**	**8**	**9**
1. Agency	1		3						
2. Pathway	0.751^∗∗∗^	1							
3.Satisfaction w. Family	0.183	0.196^*^	1						
4. Satisfaction w. Friends	0.285^∗∗^	0.368^∗∗∗^	0.318^∗∗∗^	1					
5. Satisfaction w. School	0.128	0.194^*^	0.185^*^	0.444^∗∗∗^	1				
6. Satisfaction w. Environment	0.201^*^	0.195^*^	0.183^*^	0.215^∗∗^	0.204^*^	1			
7. Intrusion/Hyper-arousal	–0.147	–0.113	–0.115	–0.109	0.133	−0.147	1		
8. Avoidance	−0.188^*^	–0.152	–0.120	–0.118	0.085	−0.145	0.752^∗∗∗^	1	
9. Negative affect	–0.129	−0.200^*^	0.067	0.001	0.072	−0.079	0.165^*^	−0.174^*^	1

^*^p < 0.05, ^∗∗^*p* < 0.01, and ^∗∗∗^*p* < 0.001.

**FIGURE 2:**
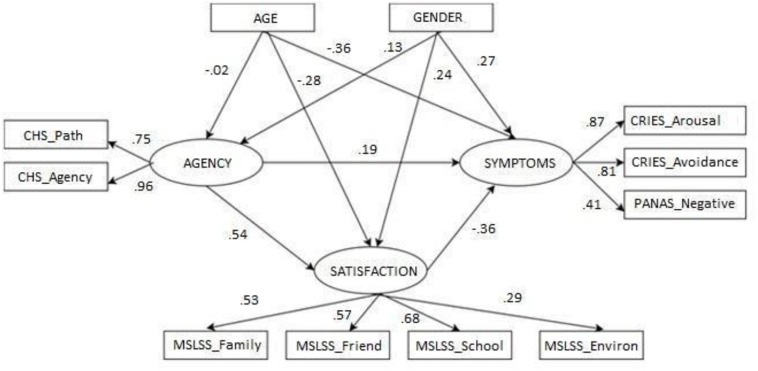
Structural model with symptoms of trauma as target variable: standardized direct effects. Results should be read from left to right. Arrows indicate direct effects between variables; ellipses indicate latent variables; and rectangular boxes represent observed variables. Age and gender were modeled as covaryingvariables.

The preliminary analysis supported the notion that agency was more strongly associated with the domains of life satisfaction and less strongly with trauma symptoms, but did not allow us to test the cumulative network of associations among the variables of interest or to break down the total effects into direct and indirect effects. In fact, the advantages of SEM models include the estimation of a regression equation to simultaneously evaluate whether the proposed model fits with the data and estimate measurement errors. Hence, the structural equation model provided a more comprehensive view (see Figure 2).

Analysis of the goodness of fit indexes supported statistical acceptance of the model, with both relative [χ2(37) = 53.1, *p* = 0.032; NC = 1.47] and absolute (RMSEA = 0.041, 95% CI = 0.012–0.163, NFI = 0.932, NNFI = 0.977, CFI = 0.976) indicators attaining values above the suggested thresholds. With regard to the pathway analysis, children’ sense of agency was found to wield direct positive standardized effects on life satisfaction (β = 0.54, *p* < 0.001) and trauma symptoms (β = 0.19, *p* < 0.05). The direct effect of life satisfaction on symptoms of trauma was statistically significant and negative (β = −0.36, *p* < 0.001). When the total effects were broken down, the indirect effect of children’s agency (via life satisfaction) was negative and statistically significant (β = −0.20, *p* < 0.01), suggesting that sense of agency may be involved in lessening the consequences of trauma by impacting on children’ feelings of life satisfaction. In addition, sense of agency wielded positive indirect effects on all domains of children’ satisfaction, with values ranging between β = 0.37 (i.e., satisfaction with school; *p* < 0.01) and β = 0.16 (i.e., satisfaction with environment; *p* < 0.05).

Interestingly, the indirect effect of life satisfaction on trauma symptoms was greater in the case of intrusion/hyper-arousal (β = −0.29, *p* < 0.01) and avoidance (β = −0.31, *p* < 0.01) and smaller for negative affect (β = −0.14, *p* < 0.05).

Children’s gender appeared to be mainly associated with trauma symptoms (β = 0.27, *p* < 0.05) and life satisfaction (β = 0.24, *p* < 0.05), with boys reporting higher levels of both than girls. Similarly, children’s age wielded direct, significant and negative effects on both life satisfaction (β = −0.28, *p* < 0.05) and trauma symptoms (β = −0.36, *p* < 0.05).

In order to further investigate the associations among the variables, we created two cohorts of children based on their “generator of agency” (sense of agency) scores, obtained by computing an aggregate score for agency, and pathway. The sample was then split in two by applying the quartile method (i.e., the 25 and 75th percentiles were the lower and upper quartiles, respectively; Altman and Bland, 1994). Finally, analysis of variance was conducted for the two resulting cohorts to test whether there were statistical variations in any of the target variables as a function of agency. Cohen (1992) was also computed. The results are reported in Table 4.

**TABLE 4:** Analysis of variance in study variables as a function of high (H) vs. low (L) levels of agency.

	**Difference (H-L)**	***F***	***p***	***d***
1. Satisfaction with family	2.20	6.37	0.001	1.07
2. Satisfaction with friends	2.65	7.15	0.001	1.13
3. Satisfaction with school	2.02	5.21	0.001	0.87
4. Satisfaction with living environment	0.72	1.93	0.056	0.32
5. Intrusion	–0.69	0.89	0.377	–
6. Arousal	0.01	0.01	0.995	–
7. Negative affect	–1.37	2.18	0.031	0.14

*Degrees of freedom = 141; following Cohen (1992), effect sizes were considered large if *d* > 0.80, medium if *d* > 0.50, and small if *d* > 0.20.*

The analysis of variance suggested that children with high agency scores were less likely to report, with medium-large effect sizes, high levels of satisfaction with family, friends, and school.

A small, statistically significant difference, was found for negative affect scores, with higher agency associated with less negative affect. In contrast, the two groups did not differ in terms of their intrusion and arousal scores.

## Discussion

The aim of our research was to test the role of agency in improving life satisfaction among Bedouin children living in a context of ongoing political and military violence in Palestine. Moreover, we expected life satisfaction to be negatively associated with trauma symptoms and to regulate children’s negative emotions. As in previous studies, our findings confirmed the key role of life satisfaction in mitigating traumatic reactions (Veronese et al., 2012a, 2017a; Veronese and Pepe, 2017a; Varela et al., 2018). Hence, the novel finding of this pilot study was the role of agency in enhancing life satisfaction which, in turn, contributed to mitigating trauma-related symptoms. When social relations (peers and family), school and, partially, living environments, were perceived by the children as sufficiently protective and safe, this contributed to reducing trauma-related symptoms such as arousal and intrusion. In addition, good life satisfaction mitigated, and protected children from, negative emotions, improving their functioning in the aftermath of traumatic experiences (Badour et al., 2017; Heleniak et al., 2018). Indeed, higher levels of general life satisfaction were associated with reduced negative emotions and trauma symptoms (H2), despite the fact that Bedouin children are continuously exposed to ongoing traumatic events (Marey-Sarwan et al., 2018). Furthermore, the association between agency and life satisfaction (H1) suggests that agency may be viewed as contributing to enhancing life satisfaction and, consequently, as playing a role in protecting children from trauma-related symptoms. More specifically, agency directly impacted life satisfaction, mainly in the domains of family and peers (social dimension), but also directly enhanced satisfaction with participants’ school and living environment, thereby contributing, mainly in older boys, to reducing arousal and intrusion symptoms (Veronese et al., 2017a; Diab et al., 2018b). For cultural reasons, boys are more inclined to actively engage in social and community activities; on the one hand, this can promote psychological well-being but on the other hand, it can increase boys’ exposure to traumatic and violent experiences (Punamäki et al., 2015; Diab et al., 2018a). Boys are also given more freedom to act on their living environment, spending more time on the streets and demonstrating against the occupation than girls, who are typically more sheltered, spending more time in safer domestic environments and engaging less frequently in social activities (Veronese et al., 2017b). Girls displayed lesser agency and higher levels of trauma-related symptoms. Young children were less likely to be agentic. However, life satisfaction proved to be an essential, though not sufficient, resource for controlling trauma. Indeed, the direct and positive relationship found between agency and trauma clearly showed that agency alone is not enough to protect children from trauma when they are not satisfied with their lives. On the contrary, when children are active but not in terms of gaining control over their life satisfaction, they are even more at risk of developing trauma symptoms and increased negative affect. These preliminary results suggest that agency without the indirect effect via life satisfaction should be viewed as a risk factor that exposes children, especially boys, to trauma symptoms and increased negative affect.

## Conclusion

Our findings help to confirm that children’s agency plays a key role in promoting their psychological wellbeing in settings characterized by political violence and systematic discrimination (Graf and Schweiger, 2017). For children in these situations, possessing agency means having a sense of control and power over their own lives, a self-perceived and expressed capability to act on and transform internal (emotional and psychological) and external (behavioral, relational, and environmental) dynamics causing psychological distress and unwellness associated with the ongoing violence to which they have been chronically exposed in a specific historical period and context (Watters, 2014). School, living environment, family, and peer relations are the domains in which children deploy agency to protect themselves from extreme trauma (Stoecklin, 2018; Veronese et al., 2018). In the case of the Bedouin children in this study, living in a hostile environment and yet maintaining good life satisfaction helped to maintain satisfactory psychological functioning in the wake of trauma.

Thus, psychological and psychosocial interventions must take into account the Bedouin child’s dual nature as a socially and politically situated actor who is actively engaged in the daily struggle for resistance and existence (Marshall, 2016). Narrative and participatory instruments can help children to transform their daily actions and habits into healing strategies and protective narratives for coping with chronic trauma and violence (Veronese et al., 2010; Marshall and Sousa, 2017; Veronese and Barola, 2018). Esthetic, reflective, and dialogical practices must be oriented toward activating and reinforcing children’s transformative capabilities, helping them to become agentic in a context that tends to victimize and passivize (Marshall, 2013). Accordingly, the fact that traditional psycho-biological and medically oriented psychotherapeutic models tend to psychiatrize child victims, underestimating their natural survival skills and resilience, can cause iatrogenic effects when children display the ability to resist in a hostile, life-threatening, and uncertain context (Gilligan, 2009; Rabaia et al., 2014).

Some critical discussion is warranted concerning the role of negative emotions in our proposed modeling of stress in this group of Bedouin children. In previous studies carried out with child victims of war and extreme violence, negative emotions were found to moderate the relationship between life satisfaction and trauma, providing evidence for a model of subjective well-being (Diener, 1984) as a moderator of trauma (Veronese et al., 2012a, 2017a). However, in the literature as a whole, the nature of the association between affect and trauma remain unclear (Chemtob et al., 1997; Kubany and Watson, 2002; Monson et al., 2004; Spitzer et al., 2007). Different studies have conceptualized negative emotions as either predictors of trauma or a symptom of traumatic stress (Veronese et al., 2017a; Knefel et al., 2018; Khamis, 2019). On the one hand, positive affect appears to be an independent variable that is not correlated with trauma symptoms; on the other hand, weak, though statistically significant correlations have been found between negative affect and life satisfaction, as well as between negative affect and trauma (Veronese et al., 2017a). This prompts us to consider the possibility that affect, as suggested by the DSM V (Brown et al., 2016), may be an outcome variable rather than a precursor of trauma. Thus, in the current pilot study, we introduced negative affect among the set of trauma-related symptoms in our model. However, some doubt remains about the role of negative emotions in traumatic situations. It is plausible to surmise that negative emotions, as conceptualized in Diener (1984) model of subjective well-being, have the power to moderate the relationship between life satisfaction and trauma up to a certain magnitude of traumatic response; but that when the level of trauma increases from moderate to high, negative emotions may be thought of as symptoms in their own right (American Psychiatric Association [APA], 2013; Badour et al., 2017). This is akin to hypothesizing a bi-directional association between negative emotions and trauma. From a clinical perspective, it is crucial to determine when negative emotions may be considered predictors, and when they can turn into symptomatic reactions. Clinical observation seems to suggest that in conditions of extreme and prolonged trauma, some negative emotions are symptomatic (Villalta et al., 2018; Khamis, 2019). In any case, this relationship needs to be further investigated in randomized studies, both longitudinally and with clinical groups of traumatized children. Age, gender, and cultural variables should also be taken into account when studying the associations between emotion and trauma (Kucharska, 2018; Khamis, 2019).

### Limitations

The limitations of the present research should be noted. First, the operationalization of agency via the CHS (Snyder et al., 1991, 1996) is unsatisfactory because it fails to capture the full complexity of the construct. The measures of agency and pathways are very broad and thus insensitive to setting-specific dimensions or sources of agency in the environment of political and military violence in which these children are growing up. New *ad hoc* and culturally sensitive instruments would facilitate a fuller operationalization of the construct of agency in clinical psychology, and in a Palestinian context characterized by ongoing trauma and structural violence. Nonetheless, the instrument we used in our pilot study, the *CHS* (Snyder et al., 1997a, b), has previously been widely used in the region where our pilot research was carried out. In a study with over 1,500 Palestinian children living in settings comparable to the ones in which the current participants are growing up, the instrument was found to display satisfactory psychometric properties, including internal consistency, and 1-month test–retest validity. In addition, the scale exhibited convergent, discriminant, and incremental validity, including significant negative correlations with depression and significant positive correlations with social competence (Khamis, 2013). Furthermore, the *CHS* also displayed good reliability in a study in which it was completed by 950 Israeli children of Palestinian/Arabic origin and a total of over 2000 participants, both Arab and Jewish, living in Israel (Kaye-Tzadok et al., 2019).

With regard to internal consistency, the estimated values were generally satisfactory, except for two dimensions of life satisfaction (satisfaction with friends and satisfaction with the environment). This discrepancy may have been due to the fact that in measuring different domains of life satisfaction, the assumption of tau-equivalence (i.e., all factor loadings are the same; Graham, 2006; Pepe et al., 2017) was violated. In addition, large standardized beta weights may be an artifact of common method variance (CMV; i.e., variance that is attributable to the measurement method rather than to the constructs themselves, see Podsakoff et al., 2003). Finally, the cross-sectional nature of our research design prevents us from generalizing from our results; future research will be focused on measuring trajectories of agency over time in diverse populations of children affected by chronic and structural violence, and on identifying the predeterminants of agency and their role in mitigating trauma symptoms and thereby enhancing children’s mental health and subjective wellbeing. The cross-sectional nature of the study and the fact that it did not use a randomized or experimental research design also means that we cannot infer causal relations among the variables. The statistical performance of the tested model suggests a chain of associations, whereby dimensions of life satisfaction and agency are associated with symptoms of trauma. Although the results of structural models have often been discussed in terms of causality (Bollen and Pearl, 2013), we agree with the position advanced by Pearl (2012). Accordingly, causal effects in observational studies “can only be substantiated from a combination of data and untested theoretical assumptions, not from the data alone” (Pearl, 2012; p. 2). Furthermore, causation cannot be strongly claimed in the absence of manipulation. In our cross-sectional study, the variables were assessed at only one time point (T1). At present, we are re-collecting the data (T2) in order to longitudinally test the directionality of associations among the variables and examine potential predictive effects.

### Final Remarks

Our study falls within the “salutogenic” paradigm (Antonovsky, 1996), according to which both individual and contextual factors play a crucial role in boosting mental health, and reducing trauma symptoms and negative emotions. Research like ours with non-clinical samples can shed light on psychological functioning and adaptation in populations experiencing ongoing, cumulative hardship. Instead of assuming that children’s psychological reactions to certain extremely violent events are invariably abnormal, such studies can help to answer more nuanced questions about the phenomenon, such as when a potentially traumatic event may not produce pathological responses (Summerfield, 1999, 2001). Thus, studying populations that have not developed symptoms in the aftermath of war and extreme violence, can provide the clinical sciences with meaningful insights about protective and adaptive factors among the child victims of war and political violence (Barber, 2014). Within this conceptual framework, we view children’s agency and life satisfaction as deep-seated and structural individual dimensions, similar to the domains of internal quality of life theorized by Jahoda (1958). These slow-changing deep structures interact with environmental factors to shape individual experience. Trauma symptoms and negative emotions (as well as other transitory mental states) on the other hand are the active outcome of the interaction between individuals and their environment.

Finally, as a further future direction of inquiry, the current absence of culturally sensitive instruments for assessing the role of agency in association with life satisfaction and trauma leads us to suggest that this construct should be explored using more accurate, and context-specific measures. Such measures should be developed using qualitative and mixed method exploratory research designs, with the potential to build up an in-depth picture of the domains of agency in Bedouin children living under political oppression (Veronese et al., 2017b, 2018).

In conclusion, the outcomes of the present pilot quantitative stage of our research program encourage us to pursue even greater understanding of the role of agency in reinforcing life satisfaction and controlling trauma. All the more so because trauma in war-affected regions is generally not a matter of episodic traumatic events, but rather is continuous and insidious and thus, potentially, more difficult to overcome. Hence, in settings characterized by ongoing trauma it is crucial to study the factors that can foster resilience and boost psychological functioning, particularly in vulnerable groups such as children.

## Ethics Statement

University of Milano-Bicocca ethic committee. Written informed consent was obtained both from children and their parents.

## Author Contributions

GV planned the research and methods, and revised the analysis. AP did the statistical analysis. GV, AP, and FC wrote the manuscript. HA and JP collected the data and revised the manuscript. All authors approved the final version of the manuscript.

## Conflict of Interest Statement

The authors declare that the research was conducted in the absence of any commercial or financial relationships that could be construed as a potential conflict of interest.
